# HETEROGENEOUS EFFECTS OF CHILDHOOD ADVERSITY ON LATE-LIFE BRAIN HEALTH: A CAUSAL FOREST APPLICATION

**DOI:** 10.1093/geroni/igae098.3210

**Published:** 2024-12-31

**Authors:** Eleanor Hayes-Larson, Ryo Ikesu, Kosuke Inoue, Paola Gilsanz, Rachel Whitmer, M Maria Glymour, Dan Mungas, Elizabeth Rose Mayeda

**Affiliations:** USC Leonard Davis School of Gerontology, Los Angeles, California, United States; University of California Los Angeles, Los Angeles, California, United States; Kyoto University, Kyoto, Kyoto, Japan; Kaiser Permanente Northern California, Oakland, California, United States; University of California Davis, Davis, California, United States; Boston University, Boston, Massachusetts, United States; University of California Davis, Davis, California, United States; University of California Los Angeles, Los Angeles, California, United States

## Abstract

Effect heterogeneity across individuals may help explain inconsistent evidence regarding effects of childhood adversity on brain health. We used harmonized Kaiser Healthy Aging and Diverse Life Experiences and Study of Healthy Aging in African Americans (n=617) data to evaluate heterogeneity in effect estimates of childhood adversity (z-score of 7 adverse childhood events factor score, dichotomized at median) on brain white matter hyperintensity volume (WMH, log transformed). We used an honest causal forest with augmented inverse probability weighting and 10-fold cross-validation to estimate conditional average treatment effects (CATEs); this approach captures complex heterogeneity better than traditional regression models. Candidate sources of heterogeneity included age at MRI, demographics, US southern birth, childhood SES measures and interactions between variables. Overall, exposure to at least median childhood adversity was associated with 0.14 higher log units of WMH (95% CI -0.06, 0.35) after covariate adjustment. The best linear fit model for the observed treatment effect had an out-of-bag predicted treatment effect coefficient of 0.81 (P-value=0.02), indicating the heterogeneity in the association. Individuals with estimated CATEs below the median estimated CATE were older (mean age 76.4y vs. 72.5y), more likely to be male (56% vs 63%), and more likely to report low childhood SES (55% vs. 74% average/well-off, 16% vs 7% ever went hungry). This is preliminary work in a relatively small sample; more work is needed to understand the impact of childhood adversity on late-life brain health, including differences across individual characteristics.

